# Supplementary material for: Predicting visual function by interpreting a neuronal wiring diagram
Source: Nature. 2024 Oct 2;634(8032):113–23. doi: 10.1038/s41586-024-07953-5 (PMC11446822; doi:10.1038/s41586-024-07953-5)
Supplement: Supplementary file 6 — CRF and ERF predictions for individual TmY4 and TmY9 cells. Analogous to Supplementary Data 3, but for TmY target types. Shown are the top four monosynaptic pathways, the strongest pathway passing through each of the top ten intermediary types (ranking from Extended Data Fig. 7), and the trisynaptic pathway Tm1–TmY–Dm3–TmY (see the section entitled Prediction of spatial normalization). [file 41586_2024_7953_MOESM6_ESM.zip › DataS4/TmY9qΓèÑ/720575940625997863.pdf]

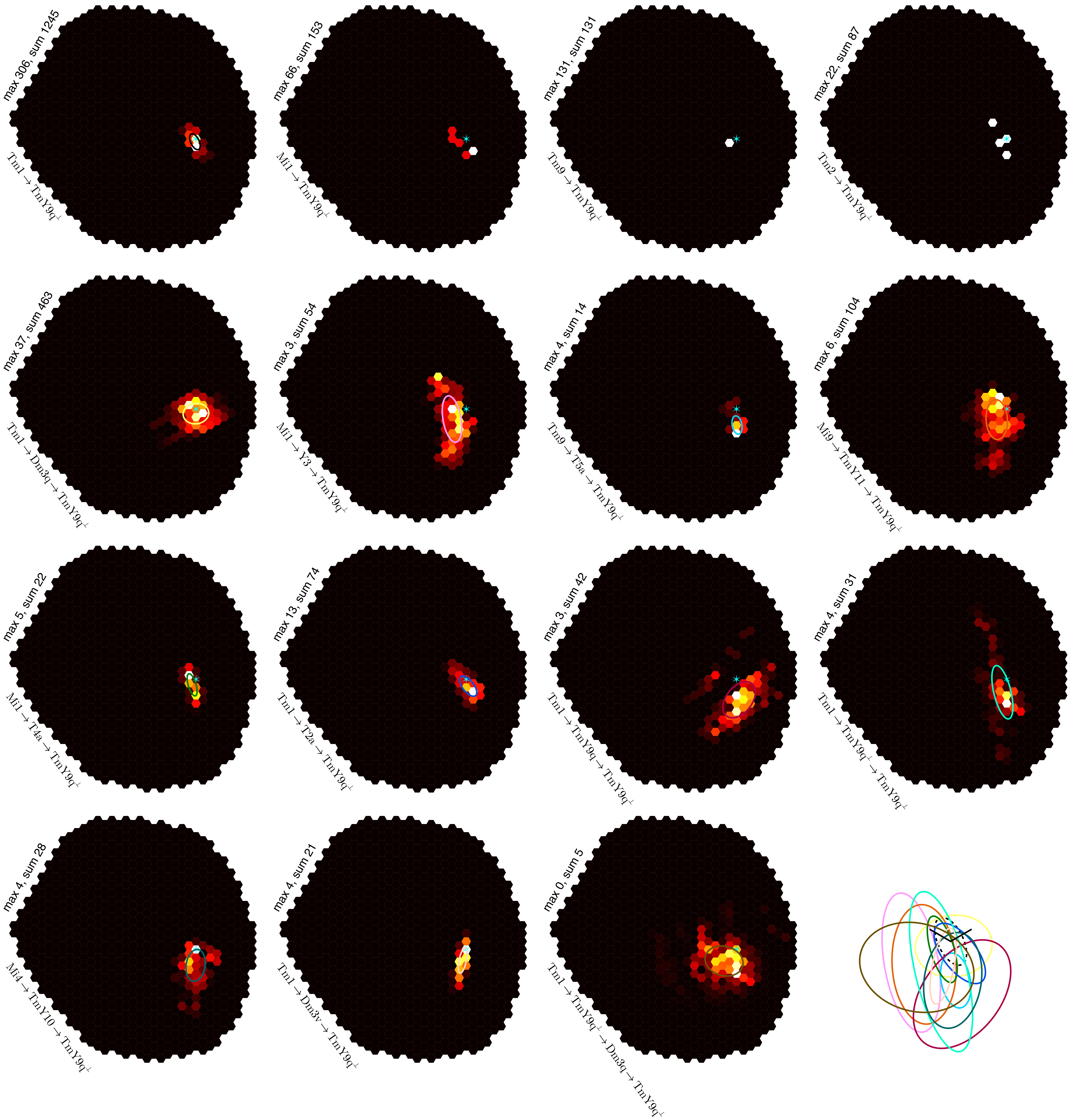
max 306, sum 1245
Tm1 → TmY9q⊥
max 66, sum 153
Mi1 → TmY9q⊥
max 131, sum 131
Tm9 → TmY9q⊥
max 22, sum 87
Tm2 → TmY9q⊥
max 37, sum 463
Tm1 → Dm3q → TmY9q⊥
max 3, sum 54
Mi1 → Y3 → TmY9q⊥
max 4, sum 14
Tm9 → T5a → TmY9q⊥
max 6, sum 104
Mi9 → TmY11 → TmY9q⊥
max 5, sum 22
Mi1 → T4a → TmY9q⊥
max 13, sum 74
Tm1 → T2a → TmY9q⊥
max 3, sum 42
Tm1 → TmY9q → TmY9q⊥
max 4, sum 31
Tm1 → TmY9q⊥ → TmY9q⊥
max 4, sum 28
Mi4 → TmY10 → TmY9q⊥
max 4, sum 21
Tm1 → Dm3v → TmY9q⊥
max 0, sum 5
Tm1 → TmY9q⊥ → Dm3q → TmY9q⊥
